# Oxidoreductase disulfide bond proteins DsbA and DsbB form an active redox pair in *Chlamydia trachomatis*, a bacterium with disulfide dependent infection and development

**DOI:** 10.1371/journal.pone.0222595

**Published:** 2019-09-19

**Authors:** Signe Christensen, Maria A. Halili, Natalie Strange, Guillaume A. Petit, Wilhelmina M. Huston, Jennifer L. Martin, Róisín M. McMahon

**Affiliations:** 1 Institute for Molecular Bioscience, University of Queensland, St Lucia, Queensland, Australia; 2 Griffith Institute for Drug Discovery, Griffith University, Nathan, Queensland, Australia; 3 School of Life Sciences, University of Technology Sydney, Broadway, New South Wales, Australia; University of the Pacific, UNITED STATES

## Abstract

*Chlamydia trachomatis* is an obligate intracellular bacterium with a distinctive biphasic developmental cycle that alternates between two distinct cell types; the extracellular infectious elementary body (EB) and the intracellular replicating reticulate body (RB). Members of the genus *Chlamydia* are dependent on the formation and degradation of protein disulfide bonds. Moreover, disulfide cross-linking of EB envelope proteins is critical for the infection phase of the developmental cycle. We have identified in *C*. *trachomatis* a homologue of the ***D***i***s***ulfide ***B***ond forming membrane protein *Escherichia coli (E*. *coli)* DsbB (hereafter named CtDsbB) and—using recombinant purified proteins—demonstrated that it is the redox partner of the previously characterised periplasmic oxidase *C*. *trachomatis* Disulfide Bond protein A (CtDsbA). CtDsbA protein was detected in *C*. *trachomatis* inclusion vacuoles at 20 h post infection, with more detected at 32 and similar levels at 44 h post infection as the developmental cycle proceeds. As a redox pair, CtDsbA and CtDsbB largely resemble their homologous counterparts in *E*. *coli*; CtDsbA is directly oxidised by CtDsbB, in a reaction in which both periplasmic cysteine pairs of CtDsbB are required for complete activity. In our hands, this reaction is slow relative to that observed for *E*. *coli* equivalents, although this may reflect a non-native expression system and use of a surrogate quinone cofactor. CtDsbA has a second non-catalytic disulfide bond, which has a small stabilising effect on the protein’s thermal stability, but which does not appear to influence the interaction of CtDsbA with its partner protein CtDsbB. Expression of CtDsbA during the RB replicative phase and during RB to EB differentiation coincided with the oxidation of the chlamydial outer membrane complex (COMC). Together with our demonstration of an active redox pairing, our findings suggest a potential role for CtDsbA and CtDsbB in the critical disulfide bond formation step in the highly regulated development cycle.

## Introduction

Disulfide bonds add structural bracing to proteins, conferring rigidity and stability. The presence or absence of such structural disulfide bonds varies markedly by cellular compartment. In both bacteria and eukaryotes, structural disulfide bonded proteins are rarely found in the cytoplasm, but instead are prevalent in more oxidising environments. In prokaryotes, disulfide bond proteins are thus found in the periplasm, membrane, and among those proteins secreted into the extracellular environment [1]. This distribution of disulfide-bonded proteins reflects the necessity for additional robustness among these proteins to withstand environmental stresses (e.g. extreme pH, ionic stresses, proteases etc). Many of these disulfide bonded proteins play a role in bacterial pathogenicity. For example, disulfide bonds are required for the function and activity of adhesins [2], proteases [3], toxins [4] and other virulence factors [5, 6] (reviewed in [7] and [1]). Accordingly, bacteria in which members of the protein disulfide oxidative pathway have been deleted show disrupted virulence phenotypes *in vitro* [7–11] and are attenuated in several mouse models of infection [12–14].

For *Chlamydia* spp, disulfide bonding has additional significance; as well as being required for infectivity [15], disulfide bonding of the outer membrane proteins is cyclically regulated during the bacteria’s unique biphasic developmental cycle [15–19] (and reviewed in [20]). Briefly, members of the genus *Chlamydia* alternate between two distinct cell types: the extracellular infectious elementary body (EB) and the intracellular replicating reticulate body (RB). The *Chlamydia* EB is rigid, as a result of substantial disulfide bond cross linking within and between proteins associated with the outer membrane. Collectively these proteins are the Chlamydia Outer Membrane Complex (COMC), comprised of the Major Outer Membrane Protein (MOMP), two cysteine rich proteins (OmcA and OmcB), the polymorphic membrane proteins (Pmps) (except PmpD), PorB, OprB, Pal, Omp85, CTL0887, CTL0645 and the type III secretion system components CdsC, CdsD and CdsF. The COMC proteins in the EB envelope are heavily cross-linked with disulfides and this feature is critical for the infectious phase of the bacteria [17–19, 21–23].

Disulfide bond protein A (DsbA) is the primary oxidase in the disulfide oxidative pathway of bacteria [24]. DsbA catalyses the introduction of disulfide bonds into reduced and folding proteins in concert with a membrane protein partner DsbB. DsbB uses a quinone cofactor as an electron acceptor, and together the DsbA-DsbB pair ultimately shuttle electrons from a reduced protein substrate to molecular oxygen via the respiratory pathway [25, 26].

*Escherichia coli* DsbA (EcDsbA) was the first DsbA enzyme to be structurally and functionally characterised and serves as the canonical model for DsbA enzymes [24, 27, 28]. Briefly, this classic DsbA structure consists of a thioredoxin domain and an inserted α-helical bundle domain. The enzyme’s active site is a Cys-Xaa-Xaa-Cys motif located at the N-terminus of helix H1. The sequence of the Xaa-Xaa dipeptide modulates the redox character of the enzyme [29]. The catalytic surface of the protein features 3 loops: Loop 1, (linking helix H1 and β-strand B3), Loop 2 (linking helix H6 and β-strand B4) which contains a highly conserved *cis*Pro residue, and Loop 3 (linking helix H7 and β-strand B5.) Together these loops govern the enzyme’s redox properties and its interactions with protein substrates (reviewed in [30] and [31]).

Structural and biochemical characterisation of a library of bacterial DsbA proteins has revealed that although DsbA proteins from diverse bacterial species share the core structure described for EcDsbA, they exhibit variations in topology and surface features, resulting in structural differences and a nuanced range of redox character and reactivity [31]. This led to description of a classification system of DsbA proteins in which the proteins are broadly divided into two groups: DsbA-I and DsbA-II [31].

Within the DsbA-II group of DsbA proteins, four proteins have been reported that have a second, non-catalytic disulfide, in addition to the enzymatically critical active site disulfide. These are DsbA proteins from *Pseudomonas aeruginosa* (PaDsbA2 [32]), *Wolbachia pipientis* (WpDsbA1 [33]), *Mycobacterium tuberculosis* (MtbDsbA [34, 35]) and *Chlamydia trachomatis* (CtDsbA [36]). In each case, this non-catalytic disulfide staples H3 and H5 of the inserted alpha-helix domain. The functional significance of this second bond appears to be variable, with evidence for a potential regulatory role, and influence on redox potential differing between the different enzymes studied to date [32, 33]. While CtDsbA shares common structural and biochemical features with the DsbA-II group of DsbA proteins, especially, CtDsbA shares just 15% sequence identity with the canonical EcDsbA protein and approximately 20% sequence identity with other members of the structurally characterised DsbA-II class proteins that contain a second disulfide [36].

We have previously characterised CtDsbA and shown it to be mildly oxidising (redox potential of -220 mV, compared to that of -120 mV for EcDsbA [36]). Given the importance of disulfide crosslinking of the *Chlamydia* envelope in infection and *Chlamydia* development, we sought to explore the potential role of CtDsbA in chlamydial envelope disulfide bonding. We further sought to query the role of the second non-catalytic disulfide in CtDsbA and identify and characterise CtDsbA’s partner protein in *C*. *trachomatis*.

## Results

### CtDsbA is expressed early in the *Chlamydial trachomatis* developmental cycle

**Fig 1:**
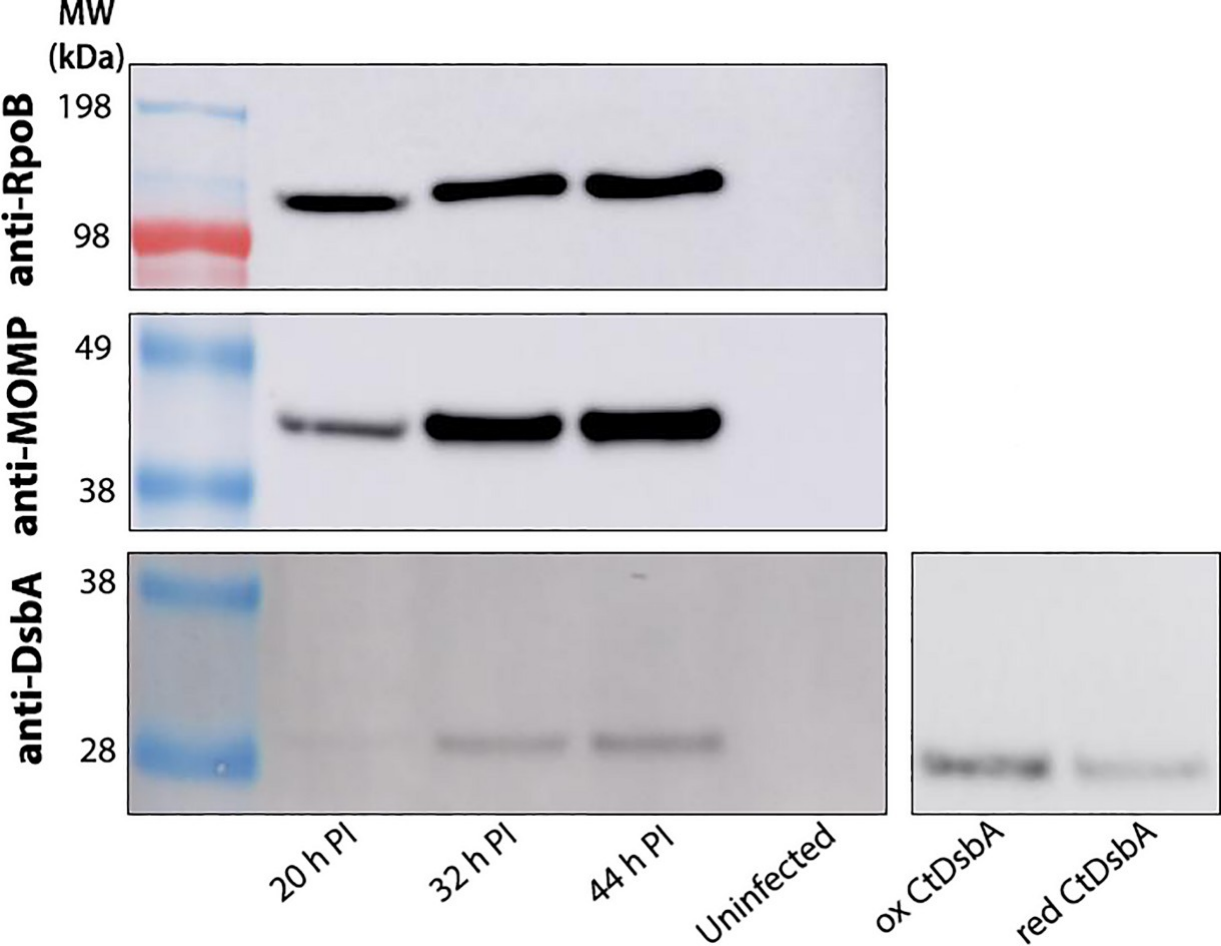
Detection of DsbA protein in *C*. *trachomatis* by Western blot. McCoy B cells were infected with *C*. *trachomatis* and harvested 20 h, 32 h and 44 h post infection (h PI). CtDsbA was detected by Western blot with an anti-CtDsbA antibody raised in rabbit and a horseradish peroxidase linked secondary antibody (lower left panel). Purified recombinant CtDsbA protein was probed with the same antibodies for reference (right panel). Detection of *Chlamydia* loading control proteins RpoB and MOMP are also shown (upper two panels). The molecular weight (MW) of the protein standards is indicated in kilodaltons (kDa).

**Fig 2:**
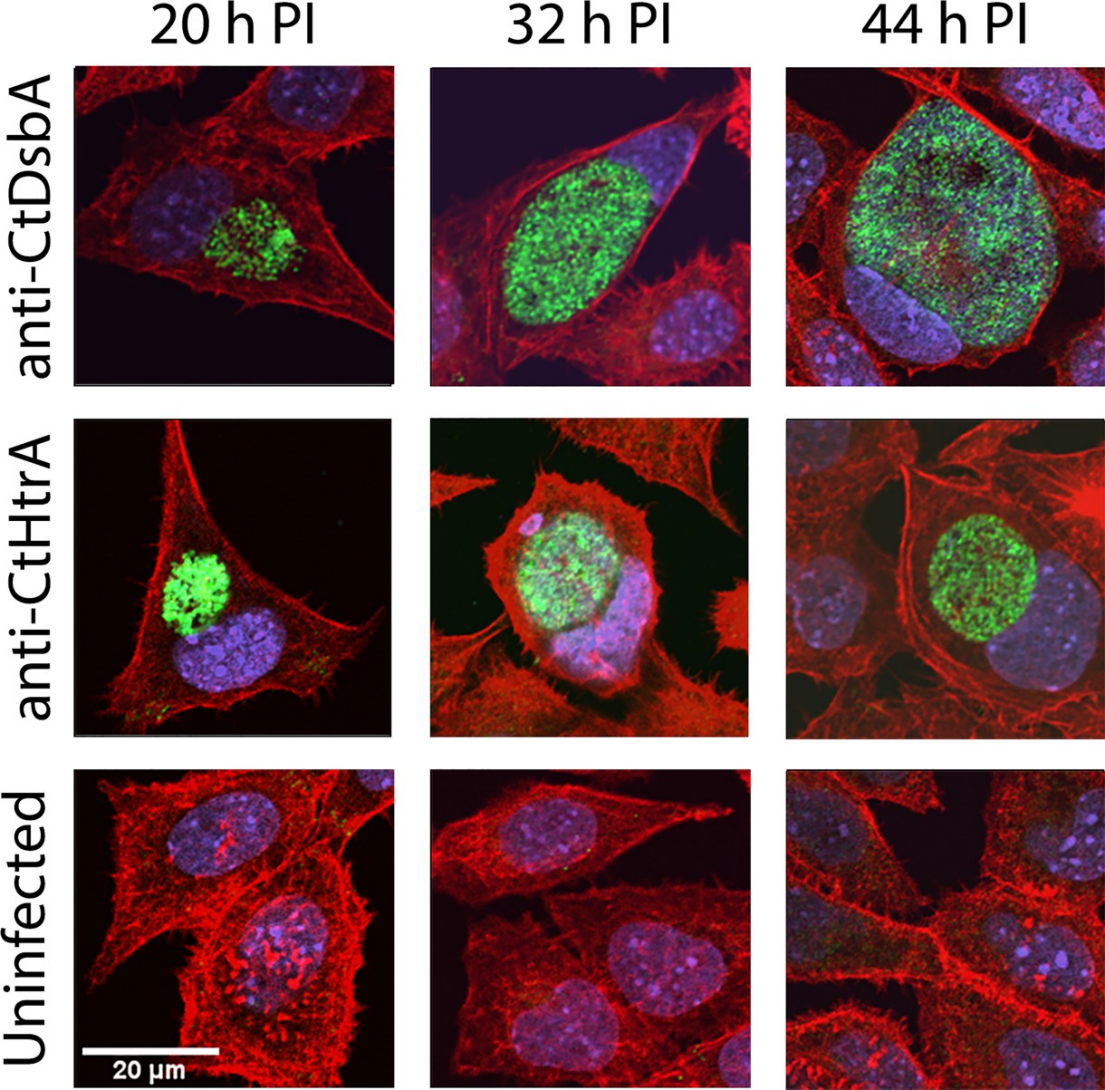
Detection of DsbA protein in *C*. *trachomatis* by confocal microscopy. Confocal images were obtained of *C*. *trachomatis* infected McCoy B cells harvested at 20 h, 32 h and 44 h post infection (h PI), in addition to an uninfected control. CtDsbA and CtHtrA are stained with anti-CtDsbA and anti-CtHtrA antibodies respectively raised in rabbit, with a secondary goat anti-rabbit IgG—Alexa Fluor 488 (green). The host cell nucleus is stained with the fluorescent dye DAPI (blue) that binds strongly to A-T rich regions in DNA. Host cell cytoskeleton is stained with Alexa Fluor 594 Phalloidin (red) that has high affinity for F-actin.

As an initial exploration of the biological role of CtDsbA, we first investigated when in the chlamydial developmental cycle CtDsbA is expressed. Using an antibody generated using recombinant CtDsbA as an antigen, we monitored the expression levels of CtDsbA protein following infection of McCoy B cells within the *C*. *trachomatis* inclusion vacuole and whole culture extracts (Figs 1 and 2). For Western Blot analysis, antibodies targeting the major outer membrane protein (MOMP) and RNA polymerase beta (RpoB) were used to adjust the loading of whole cell lysates (WCLs) to bring levels at the typically low abundant 20 h PI time point to closer to that of later stages. CtDsbA protein was able to be clearly detected at 32 h post infection (h PI) by Western blot (Fig 1). It appears that the CtDsbA protein may also be present at 20 h PI, although the signal is faint. Confocal imaging using the same anti-CtDsbA antibody and a green Alexa Fluor labelled secondary antibody, detected CtDsbA at 20 h post infection but not in the uninfected controls, indicating that some CtDsbA protein is present at this time point (Fig 2). At 32 h and 44 h post infection the expression of CtDsbA is clearly present in the inclusion vacuoles (Fig 2). An antibody specific to *C*. *trachomatis* HtrA (CtHtrA) was additionally used as a positive labelling control, as this antibody has previously been shown to detect *C*. *trachomatis* at the infection time points analysed [37]. We note that at the 20 h stage of the chlamydial developmental cycle, RB replication is dominant and that the asynchronous conversion to elementary body EB begins around this time, including oxidation of the disulfides in the chlamydial outer membrane complex [38, 39]. Given the possible correlation between CtDsbA expression and onset of disulfide cross-linking of the proteins in the chlamydial envelope, we hypothesised that a DsbA-DsbB redox relay might be involved in the regulation of the disulfide bond status of the chlamydial outer membrane complex and sought to identify a potential DsbB protein in *C*. *trachomatis*.

### *C*. *trachomatis* encodes a homologue of DsbB

**Fig 3:**
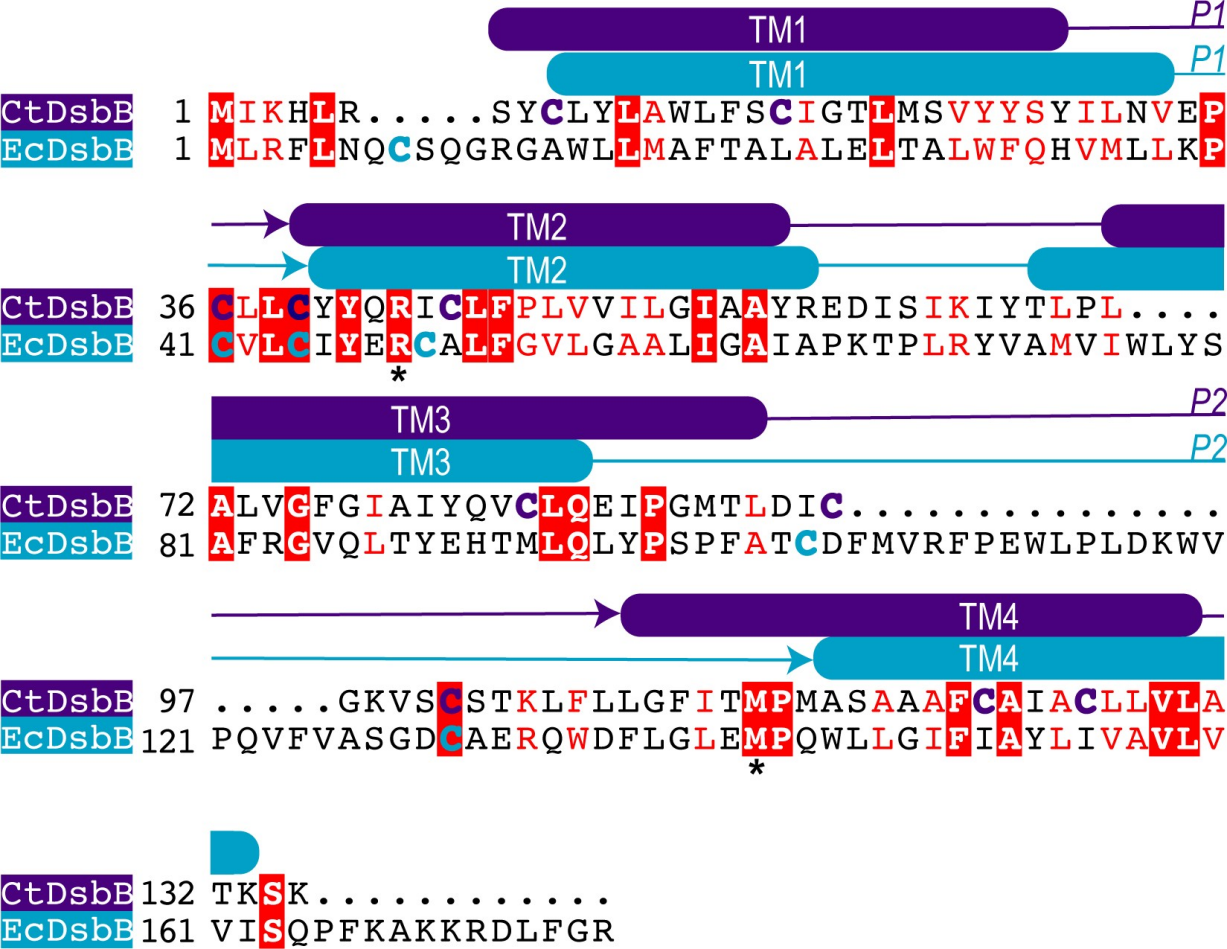
Sequence alignment of DsbB from *E*. *coli* and *C*. *trachomatis*. Sequence alignment of EcDsbB and CtDsbB performed in AlignMe [42] and visualised using ESPript [43]. Membrane topology prediction (TMHMM [44]) indicates that CtDsbB (purple), like EcDsbB (blue), has four predicted transmembrane helices, TM1-4 and two predicted periplasmic regions P1 and P2 (indicated with an arrow) each containing two cysteine residues. Annotation of predicted transmembrane boundaries was informed by TMHMM sequence analysis (for both CtDsbB and EcDsbB), and the observation of alpha helices in an available crystal structure (EcDsbB) [40]. Cysteine residues are highlighted in each sequence (purple or blue as above). Residues with strict identity are shown as filled red boxes and a white character. Residues that are highly similar (above the 0.7 equivalence threshold) are shown as red character. Residues with weakly similar properties (below the 0.7 equivalence threshold) are shown as black characters. The conserved residues Arg48 and Met142 are highlighted with an asterisk.

A protein BLAST interrogation of the *C*. *trachomatis* genome (NCBI Taxid: 813) using *E*. *coli* DsbB (EcDsbB, UniProt ID P0A6M2) as the query sequence identified a protein of 135 amino acids with 22% sequence identity to EcDsbB (Fig 3). This protein will be referred to hereafter as *C*. *trachomatis* DsbB (CtDsbB). CtDsbB is predicted to have four transmembrane helices (TM1-4) and two periplasmic loops (P1 and P2), equivalent to the topology of EcDsbB [40, 41]. However, the predicted loop P2 is significantly shortened in CtDsbB (14 residues) relative to its *E*. *coli* counterpart (49 residues) (Fig 3). CtDsbB has ten cysteine residues compared to six cysteine residues in EcDsbB. Five of the cysteines in CtDsbB are embedded in the predicted transmembrane regions and one is located in the predicted cytoplasmic N-terminal region. Similar to the arrangement of the catalytic cysteines in EcDsbB, the remaining four cysteines are located in the two predicted periplasmic loops, and at the N-terminal end of TM2. In EcDsbB, these 4 cysteines constitute two functional pairs that mediate a series of thiol-exchange reactions facilitating oxidation of EcDsbA. Conservation of Arg48 and Met142, residues proposed to be involved in ubiquinone binding in EcDsbB, suggests that CtDsbB is likewise capable of binding a similar co-factor. The similarity in sequence and topology with EcDsbB suggest that CtDsbB is a redox partner for CtDsbA.

### Membranes containing CtDsbB can sustain CtDsbA catalysed oxidation of a model substrate

**Fig 4:**
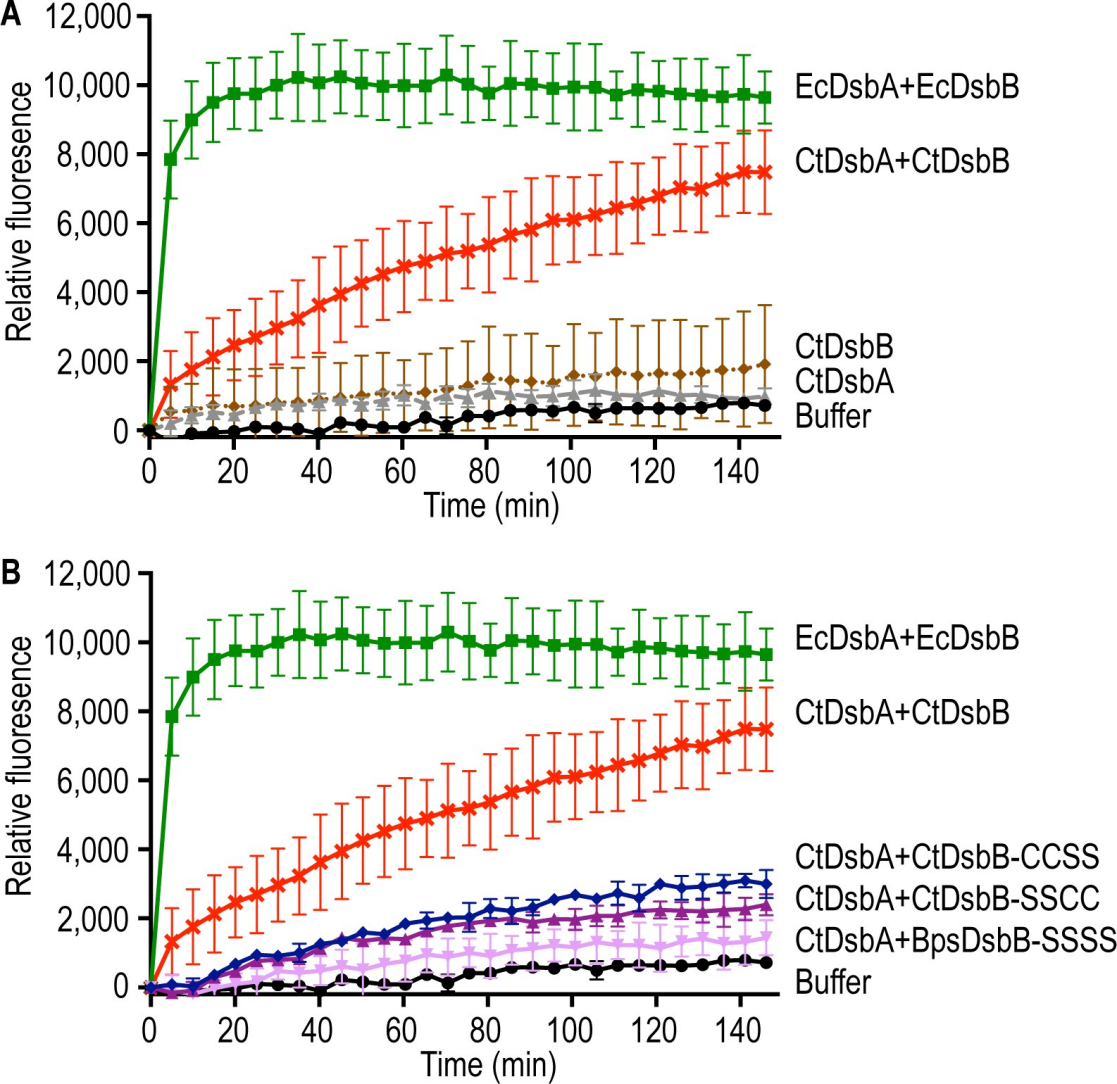
Both periplasmic disulfide bonds are required for CtDsbB to form a redox relay with CtDsbA. An increase in fluorescence as a function of time is seen as a result of CtDsbA and CtDsbB catalysed oxidation of a fluorescently labelled peptide containing two cysteines. A) A rapid increase in fluorescence is seen for 80 nM EcDsbA with membranes containing 8 μM EcDsbB (green). 640 nM CtDsbA with membranes containing 8 μM CtDsbB (red) gives a slower increase in fluorescence. Fluorescence only increases minimally for control reactions containing only CtDsbB (8 μM) membranes (brown), 640 nM CtDsbA (grey) or buffer only (black). B) The increase in fluorescence for 640 nM CtDsbA with membranes containing 8 μM CtDsbB-CCSS (blue) and 640 nM CtDsbA with membranes containing 8 μM CtDsbB-SSCC (purple) are significantly slower than 640 nM CtDsbA with membranes containing 8 μM CtDsbB (red), but marginally faster than 640 nM CtDsbA with membranes containing 8 μM BpsDsbB-SSSS (pink) and buffer only (black). The curves for 80 nM EcDsbA with membranes containing 8 μM EcDsbB (green), 640 nM CtDsbA with membranes containing 8 μM CtDsbB (red) and buffer only (black) are the same as in A. Plotted data show mean and SD for two biological replicates.

To test if CtDsbB is indeed a redox partner of CtDsbA, we recombinantly overexpressed CtDsbB in *E*. *coli*. Crude membrane extracts containing CtDsbB were assayed for their ability to sustain CtDsbA catalysed oxidation of a model peptide substrate. Briefly, in this assay purified DsbA catalyses the oxidation of an intramolecular disulfide bond in an initially reduced and unfolded peptide substrate containing two cysteine residues. Upon folding, a fluorescence signal can be measured between DOTA-europium and coumarin labels on the N and C termini, respectively, of the substrate. 8 μM CtDsbB in crude *E*. *coli* membranes successfully sustained CtDsbA (640 nM) oxidase activity (Fig 4A), although at a reduced rate relative to a positive control containing EcDsbA at an 8-fold lower concentration (80 nM EcDsbA and 8 μM EcDsbB). Essentially no oxidation of the substrate peptide was observed in control reactions containing buffer, CtDsbA, or CtDsbB alone.

In *E*. *coli* both periplasmic loop cysteine pairs of EcDsbB participate in the mechanism of EcDsbA oxidation [45–47]. To investigate whether the mechanism of CtDsbA oxidation by CtDsbB is similar to that of *E*. *coli*, two CtDsbB mutants were designed in which the cysteines in loop P1 and loop P2, respectively, are mutated to serines. In CtDsbB-SSCC, periplasmic loop 1 Cys36 and Cys39 are mutated to serines. In CtDsbB-CCSS, periplasmic loop 2 Cys98 and Cys104 are mutated to serines. In the presence of CtDsbB-CCSS or CtDsbB-SSCC, CtDsbA catalysed oxidation of the peptide substrate is markedly reduced relative to wild-type CtDsbB, although oxidation proceeds more rapidly than observed for negative controls containing only buffer, or the wild-type CtDsbB variant alone (Fig 4B). That CtDsbA is significantly less active in the presence of CtDsbB-SSCC or CtDsbB-CCSS, than in the presence of CtDsbB, suggests that as anticipated, the disulfide bonds present in periplasmic loops P1 and P2 are each required for complete oxidation of CtDsbA.

Although much less active than in the presence of CtDsbB, reactions of CtDsbA with membranes containing CtDsbB-SSCC and CtDsbB-CCSS did show some activity relative to the buffer control (Fig 4B). To exclude the possibility that other factors present in the *E*. *coli* membranes facilitate CtDsbA activity, a catalytically inactive construct of a DsbB from another organism, *Burkholderia pseudomallei* with all four cysteines in the two periplasmic loops mutated to serines (BpsDsbB-SSSS)[13] was included as a negative control (Fig 4B). Membranes containing catalytically inactive BpsDsbB-SSSS were also unable to facilitate CtDsbA activity, and most closely resembled the reaction containing buffer alone. Consequently, the observed CtDsbA activity arises from a specific interaction with CtDsbB, and other factors in the membrane preparation contribute minimally to CtDsbA activity. Taken together, this activity is consistent with CtDsbB being a redox partner protein of CtDsbA.

### Detergent solubilised, purified CtDsbB partially oxidises reduced CtDsbA

To confirm that CtDsbA oxidising activity in the model substrate assay is sustained specifically by interaction with CtDsbB in the membranes, we examined the redox state of recombinant purified CtDsbA following incubation with detergent solubilised purified CtDsbB. The redox state of CtDsbA was assessed using an electrophoretic mobility assay. Briefly, CtDsbA was treated with the thiol-reactive probe 4-Acetamido-4'-Maleimidylstilbene`-2,2'-Disulfonic Acid (AMS), which labels free thiols and adds 0.5 kDa per label to the molecular weight. This allows oxidised CtDsbA and reduced CtDsbA to be separated by SDS-PAGE, and their relative abundance quantified by densitometric analysis.

**Fig 5:**
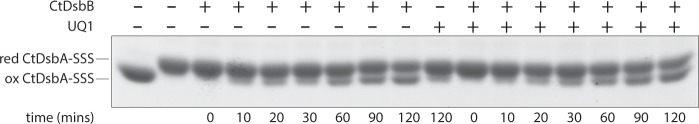
Detergent solubilised, purified CtDsbB partially oxidises CtDsbA-SSS independently of exogenous ubiquinone. The redox state of CtDsbA-SSS over time was monitored by a shift in electrophoretic mobility after treatment with the alkylating agent AMS that adds 0.5 kDa per reduced cysteine. A) Reduced CtDsbA-SSS (15 μM) was incubated with equimolar purified detergent solubilised CtDsbB (15 μM) in the absence or presence of UQ1 (15 μM) and incubated over the indicated time course. Oxidised CtDsbA-SSS is detectable after 10 min in the presence of CtDsbB (left hand side of gel) and was not affected by the addition of exogenous UQ1 (right hand side of gel). Completely reduced and oxidised CtDsbA-SSS protein samples were included on the SDS-PAGE for reference. These samples contained 5mM DTT and 5 mM oxidised glutathione respectively to keep CtDsbA-SSS reduced and oxidised throughout the 120 min incubation. Incubation of reduced CtDsbA-SSS with 15 μM UQ1 showed a small fraction of oxidised CtDsbA-SSS formed over the 120 min time course. Data presented is representative of three independent experiments from three different purifications of recombinant CtDsbA-SSS and CtDsbB, expressed following independent transformations of *E*. *coli*.

As we hypothesised that the active site CSAC thiols are the likely target for CtDsbB mediated oxidation, we first used a construct of CtDsbA in which the three non-catalytic cysteines in the protein were changed to serine (CtDsbA-SSS; [36]) Reduced CtDsbA-SSS was incubated with ubiquinone-1 (UQ1) in the presence or absence of equimolar amounts of purified, detergent-solubilised CtDsbB. After mixing, a sample was taken immediately, and after subsequent time points, as indicated in Fig 5. At each time point the reaction was stopped by precipitation with trichloroacetic acid (10% w/v) and the samples subsequently treated with AMS.

For CtDsbA-SSS incubated with CtDsbB and UQ1, the protein initially migrates at a molecular weight corresponding to reduced CtDsbA-SSS (red CtDsbA-SSS) until 10 minutes after the reaction is started, at which point CtDsbA-SSS migrates as two bands, corresponding to a mixture of oxidised (ox CtDsbA-SSS) and reduced protein. After 120 min, only half of the CtDsbA-SSS has been oxidised by CtDsbB in the presence of exogenous UQ1 (Fig 5). This reaction proceeds slowly as compared to EcDsbA and EcDsbB where in an equivalent experiment in the presence of exogenous UQ1, essentially all reduced EcDsbA is converted to the oxidised form in 10 seconds, with a minor population participating in the formation of a EcDsbA-EcDsbB complex [48]. Of note, in our hands the oxidation of reduced CtDsbA-SSS proceeds similarly in the presence and absence of exogenous UQ1, suggesting that exogenous UQ1 is not required for the reaction to proceed, although in neither case is reduced CtDsbA-SSS fully converted to oxidised over the 120 min period. Notably, when reduced CtDsbA-SSS was incubated in the presence of UQ1 alone i.e. without DsbB, a small fraction of CtDsbA-SSS was oxidised over the time course (120 min.) Taken together with the model substrate oxidation, these data suggest that CtDsbA and CtDsbB can form a functional relay, although UQ1 is likely not the ideal quinone for the reaction as evidenced by the relatively slow rates of reaction and that the redox conversion does not go to completion under the experimental conditions used.

### Unpicking the role of the second disulfide in CtDsbA

Next, we investigated the contribution of the second disulfide bond to CtDsbA’s redox character and activity. In *Wolbachia pipientis* DsbA1 (WpDsbA1), an equivalent non-catalytic secondary disulfide may have a regulatory role by autoinhibiting the interaction of WpDsbA1 with its partner *W*. *pipientis* DsbB [49], and a very modest effect on redox potential [33]. In contrast, mutation of the secondary disulfide in *P*. *aeruginosa* PaDsbA2 influences the protein’s redox potential significantly [32]. We sought to answer what role this second disulfide may play in CtDsbA, by investigating whether its removal affected CtDsbA’s interaction with CtDsbB, or altered the protein’s thermal stability of its reduced and oxidised states.

**Fig 6:**
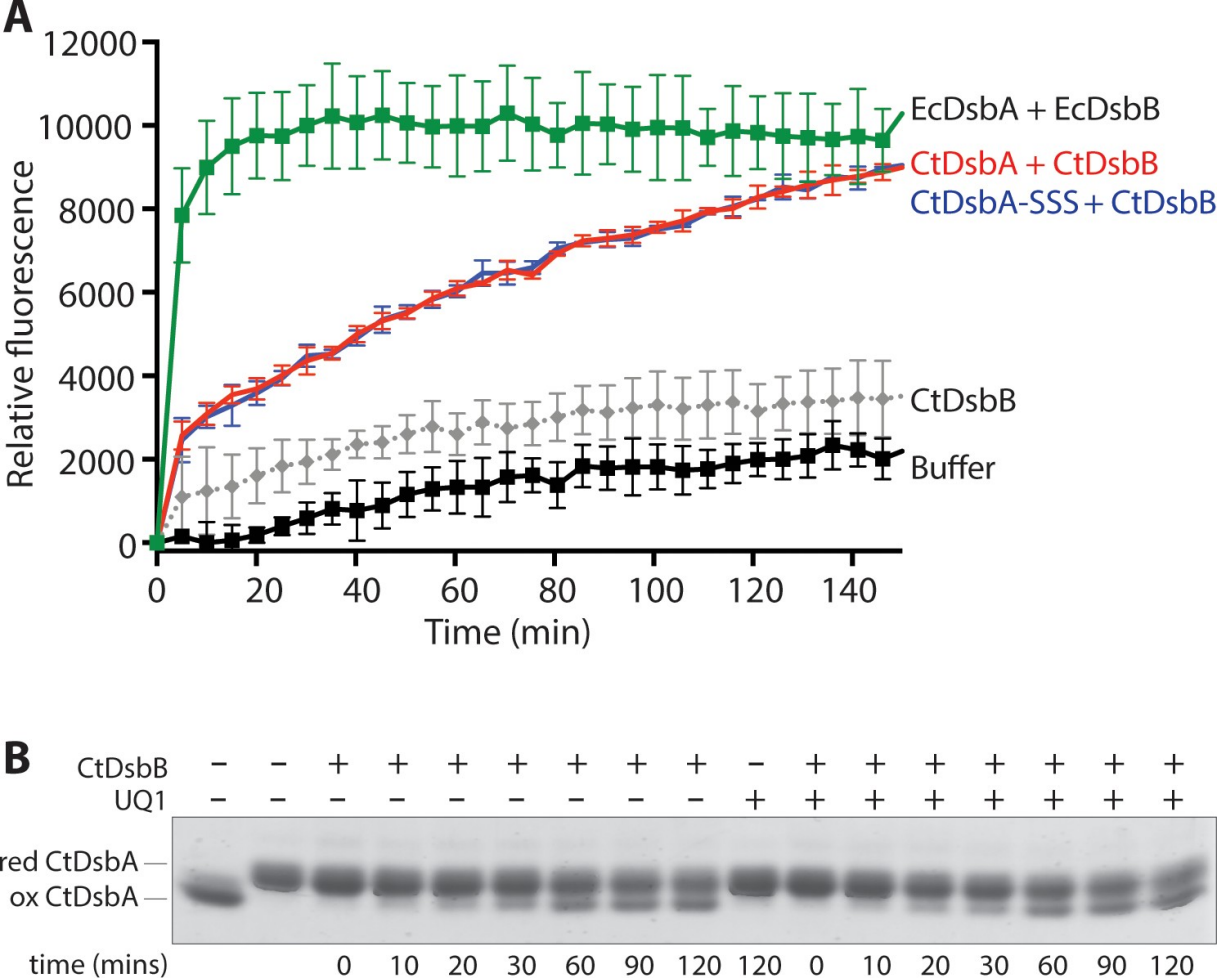
The second disulfide of CtDsbA does not influence interaction with CtDsbB. A) An increase in fluorescence as a function of time is seen as a result of CtDsbA or CtDsbA-SSS catalysed oxidation of a fluorescently labelled peptide containing two cysteines. A rapid increase in fluorescence is seen for positive control 80 nM EcDsbA with membranes containing 8 μM EcDsbB (green). The activity of 640 nM CtDsbA (red) and 640 nM CtDsbA-SSS (blue) is equivalent upon interaction with membranes containing 8 μM CtDsbB. The activity of CtDsbB (grey) is only minimally higher than fluorescence for the buffer only control (black). Plotted data show mean and SD for three biological replicates, except for the EcDsbA + EcDsbB control where data from a single experiment was used. B) Reduced CtDsbA (15 μM) was incubated with equimolar purified detergent solubilised CtDsbB (15 μM) in the absence or presence of UQ1 (15 μM) and incubated over the indicated time course. Oxidised CtDsbA was detected after 10 min in the presence of CtDsbB (left hand side of gel) and did not require exogenous UQ1 for the reaction to proceed (right hand side of gel). Completely reduced and oxidised CtDsbA-SSS protein samples were included on the SDS-PAGE for reference. These samples contained 5mM DTT and 5 mM oxidised glutathione respectively to keep CtDsbA-SSS reduced and oxidised throughout the120 min incubation.15 μM UQ1 alone was not able to oxidise CtDsbA over the 120 min time course. Data presented is representative of three independent experiments from three different purifications of recombinant CtDsbA and CtDsbB expressed following independent transformations of *E*. *coli*.

To explore the potential for the second disulfide to modulate interaction with CtDsbB, we compared the activity of CtDsbA-SSS (containing only the active site disulfide) and wild type CtDsbA (containing both the active site, secondary disulfide and an unpaired cysteine) in the model peptide substrate assay. CtDsbA-SSS and wild-type CtDsbA are equally active in their oxidation of the model substrate, suggesting that the second disulfide does not affect interaction with CtDsbB in this assay, at least not in the presence of the endogenous quinone available in the membrane preparation (Fig 6A). We note the possibility that addition of an exogenous ideal quinone may reveal reaction differences between the protein variants.

Similarly, we assessed whether CtDsbB mediated oxidation of reduced wild type CtDsbA and reduced CtDsbA-SSS proceeded equivalently using the electrophoretic mobility assay. We showed previously that detergent solubilised CtDsbB was able to oxidise CtDsbA-SSS without the addition of exogenous UQ1 (Fig 5). Here, CtDsbB was also able to oxidise reduced wild type CtDsbA in the presence or absence of exogenous UQ1 (Fig 6B), indicating that exogenous UQ1 is not required for the reaction to proceed. Once again, under these experimental conditions, a mixture of oxidised and reduced CtDsbA remained after 120 min. One difference however, was that reduced wild type CtDsbA was not readily oxidised by exogenous UQ1 alone, indicating that the oxidation reaction is predominantly mediated by CtDsbB.

**Fig 7:**
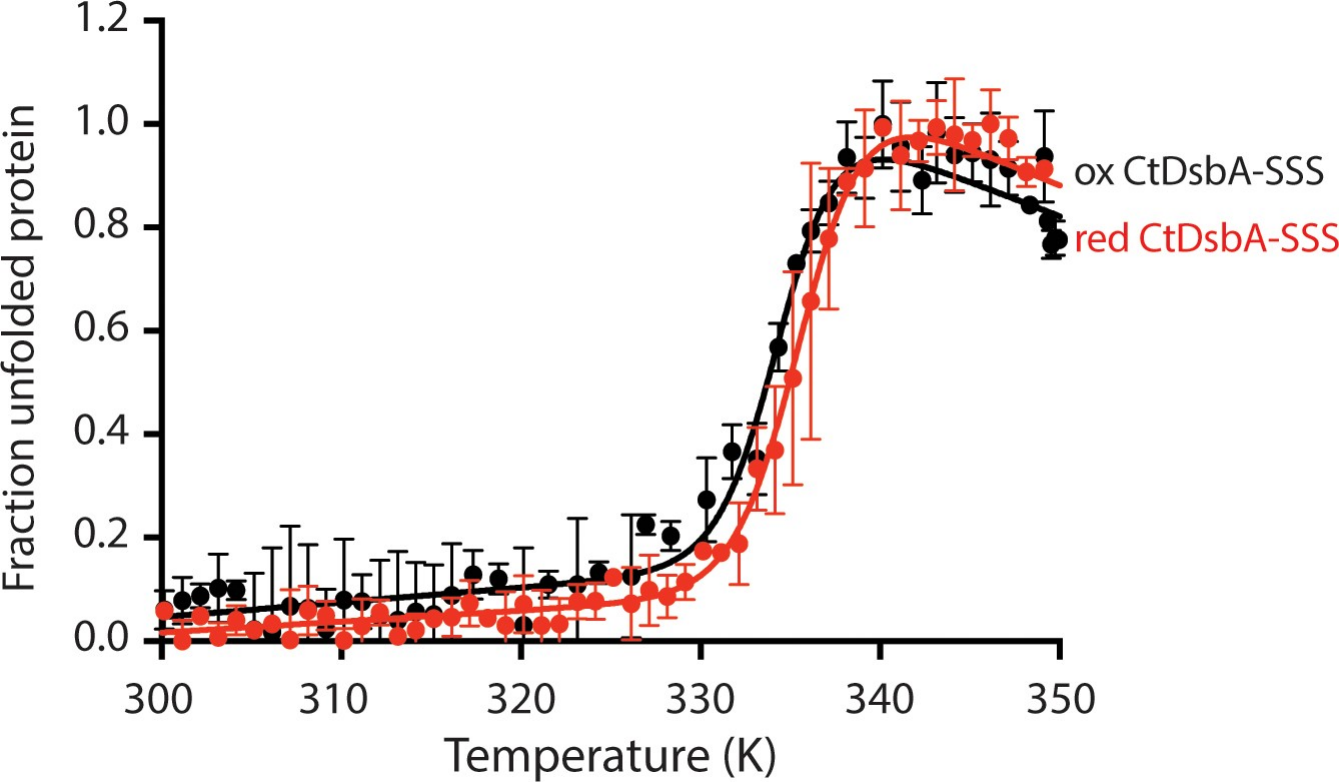
The second disulfide of CtDsbA has a limited effect on protein thermal stability. The thermal unfolding of reduced and oxidised CtDsbA-SSS was monitored by CD spectroscopy and the fraction of unfolded CtDsbA-SSS plotted as a function of temperature. With a melting temperature of 336 K ± 0.1, reduced CtDsbA-SSS is slightly more stable than oxidised CtDsbA-SSS that has a melting temperature of 334 K ± 0.2. Plotted data show mean and SD for three biological replicates.

Reduction and oxidation of disulfide bonds in proteins can change a protein’s melting temperature (Tm), i.e. the temperature at which half of the protein is unfolded. Typically, in DsbA proteins, reduction of the active site disulfide increases the melting temperature, reflecting the greater stability of the reduced form of the protein relative to the oxidised form, consistent with a thermodynamic favourability for the enzyme to oxidise substrates and itself become reduced. We have previously determined the melting temperature for reduced and oxidised CtDsbA as 339 ± 0.2 K and 335 ± 0.1 K, respectively; this is one of the smaller differential Tm values reported for DsbA proteins [36]. The melting temperature of reduced CtDsbA-SSS was determined as for wild-type using a gradual temperature ramp and monitoring protein unfolding by circular dichroism spectroscopy. The melting temperature of reduced CtDsbA-SSS was determined as 336K ± 0.1, and that of the oxidised state 334K ± 0.2 (Fig 7). As for other DsbA proteins, the melting temperature of the reduced protein is greater, indicating that the reduced state has a greater thermal stability than the oxidised state. As only the active site disulfide can form in this construct, this is consistent with the relative thermodynamically favoured reduced state of the enzyme. The difference in Tm between the two states is again modest (~2 K), and indeed smaller than that observed for wild-type protein (4 K [36]).

The reduced difference in Tm between the reduced and oxidised states of CtDsbA-SSS (ΔTm = 2 K), compared to reduced and oxidised CtDsbA (ΔTm = 4 K) suggests that the non-catalytic disulfide contributes somewhat to the relative stability of the oxidised and reduced states i.e. either by additionally stabilising the reduced form, or further destabilising the oxidised.

As reduced wild type CtDsbA (in which both active site and non-catalytic disulfides are reduced) and reduced CtDsbA-SSS (in which the active site disulfide is reduced, and the reduced second disulfide is mimicked by cysteine-to-serine mutations) both represent proteins in which both disulfides are effectively in the reduced state, it was surprising that the melting temperature for reduced CtDsbA-SSS differs from that of reduced wild type CtDsbA: 339 ± 0.2 K (reduced wild-type CtDsbA) and 336K ± 0.1 (reduced CtDsbA-SSS) (Table 1). This is most likely a result of differences in the biochemical properties of the serine compared to the reduced cysteine residue that it replaces. Together these mutations may additionally alter the overall stability of CtDsbA-SSS and contribute to the difference in melting temperature of reduced CtDsbA-SSS relative to reduced CtDsbA.

**Table 1:** Melting temperatures of CtDsbA redox states.

	Tm (K) reduced	Tm (K) oxidized	ΔTm (K)
CtDsbA	339±0.2	335±0.1	4
CtDsbA-SSS	336±0.1	334±0.2	2

The melting temperatures (Tm) of reduced and oxidised CtDsbA were determined previously [36]. Tm values for CtDsbA-SSS are from this study. Mean and SD for three biological replicates are presented.

## Discussion

We have identified a DsbB protein in *C*. *trachomatis*, and demonstrated that it is a redox partner of the previously characterised oxidase CtDsbA [36]. As a redox pair CtDsbA and CtDsbB largely resemble their homologous counterparts in *E*. *coli*: CtDsbA is directly oxidised by CtDsbB in a reaction in which both periplasmic cysteine pairs of CtDsbB are required for activity. We showed that this reaction proceeded independent of exogenously added ubiquinone, but more slowly than the EcDsbA-EcDsbB interaction and did not go to completion. Whilst these data demonstrate that CtDsbA and CtDsbB form a redox relay, they suggest that the native CtDsbB co-factor is not UQ1 or Ubiquinone-8 (UQ8) (the endogenous ubiquinone in *E*. *coli*).

A notable difference from the canonical *E*. *coli* system in *C*. *trachomatis* is the presence of a second non-catalytic disulfide bond in CtDsbA. This disulfide bond, bridging helices H2 and H5 of the protein, is conserved in DsbAs from alpha-proteobacteria including *W*. *pipientis* [49]. It is also found in the DsbA from *M*. *tuberculosis* [34] and one of the two DsbAs (PaDsbA2) encoded by *P*. *aeruginosa* [32]. In *W*. *pipientis*, this second disulfide has a modest effect on redox potential [33] but appears to inhibit the WpDsbA interaction with WpDsbB [49]. By contrast, the additional disulfide bond of PaDsbA2 does modulate the protein’s redox potential [32]. For CtDsbA, we found that this second disulfide bond has no effect on CtDsbA activity or its interaction with CtDsbB but may contribute to the relative stability of the active site redox forms of the enzyme.

Reduced EcDsbA is partially oxidised immediately upon addition of EcDsbB and UQ1, and becomes fully oxidised after 10 seconds [48]. We observe a similar trend for the *Chlamydia* proteins, supporting the observation that CtDsbA and CtDsbB are partners, but conclude also that the reaction is not optimal under the experimental conditions we used. This may reflect the fact that the proteins were expressed recombinantly in a non-native *E*. *coli* host.

Inaba *et al* have reported that a quinone-free preparation of EcDsbB oxidises ~40% of EcDsbA in a 1:1 stoichiometric reaction [48]. This may also be the case for the *Chlamydia* DsbA/B system as we found that about half of reduced CtDsbA or CtDsbA-SSS is oxidised by CtDsbB in a 1:1 ratio. The addition of exogenous UQ1 to the CtDsbB reaction had no further effect, indicating that UQ1 or endogenous UQ8 (presumed to be present in *E*. *coli* membranes), may not be its optimal cofactor.

Members of the *Chlamydia* genus are Gram negative obligate intracellular bacteria with a biphasic development cycle. In this cycle, *Chlamydia* alternates between two different cell types, the infectious extracellular EB and the intracellular RB, although this is highly asynchronous. After the infectious EBs have been internalised by host eukaryotic cells, the EBs differentiate to RBs (2–8 h post infection). The RBs subsequently replicate (8–24 h), before re-differentiating to EBs (24–72 h) in preparation for cell lysis or vacuole extrusion and release (40–72 h), ready for the next infectious event. Confocal microscopy with immunofluorescence detected CtDsbA in *C*. *trachomatis* from at least 20 h post infection onwards (also observed by Western blot). The 20 h time point represents the middle to late replicative phase of the *Chlamydia* biphasic lifecycle, when the recently differentiated RBs multiply. A previous transcriptional profiling study reported that CtDsbA gene expression begins as soon as 8 hours post infection [50], corresponding to late EB to RB differentiation and early RB replication. We were able to detect DsbA at 20 h PI using immunofluorescence microscopy, and a faint band via Western blot. The low levels of CtDsbA protein detected at 20 h post infection could arise from the use of different strains in the two studies (*C*. *trachomatis* serovar D strain UW-3/Cx in the transcriptional study compared to *C*. *trachomatis* LGV-2 used here), but more likely reflects a difference in methodology for detecting CtDsbA (transcript versus protein). Factors such as mRNA stability, protein degradation, and variable transcription and translation rates, mean that mRNA expression levels can differ by up to 60% from protein levels in eukaryotes and prokaryotes [51, 52]. A third study measured CtDsbA protein levels by label-free quantitative proteomics and found no DsbA in replicating RBs 18 h post infection, but did detect CtDsbA in EBs 44 h post infection, around the time of asynchronous exit from the host cell [53]. In our hands, CtDsbA protein levels increased markedly between 20 and 32 h PI and remained at a high level at 44 h post infection. In summary, CtDsbA protein was detectable at 20 h post infection, although CtDsbA transcription onset may precede this by up to 12 hours.

Within the *C*. *trachomatis* COMC, the MOMP is synthesised early in the replicative phase but is not oxidised until the onset of RB to EB differentiation (at approximately 24 h post infection) [22, 38, 39, 50, 54, 55]. The two cysteine-rich proteins, OmcA and OmcB are synthesised late in the replicative phase (up to 24 h post infection) but are oxidised immediately [22, 38, 39, 50, 54]. The coincidence of CtDsbA expression and the onset of COMC protein oxidation is notable, and could support the notion that the COMC is one possible substrate of CtDsbA. DsbJ and DsbH are also likely to be important contributors to the COMC protein redox status. The expansion of the number of genes encoding for oxidoreductases in *Chlamydia* is likely due to the critical role that redox status has on the developmental cycle, mediated by the COMC. Presumably given the reduced genome nature of this organism, the oxidoreductases are likely all critical. It is possible that individual proteins have specialised substrate selectivity or, given how critical the redox status of the COMC is for the biology of this organism, that there is functional redundancy, although the precise function of individual oxidoreductases in *Chlamydia* is currently unknown [20].

Disulfide-dependent infection and development is a unique feature of *Chlamydia*, though the suite of Dsb proteins in *Chlamydia* are, with the exception of DsbJ, present in other bacteria (reviewed in [20]). Continued elucidation of the intricacies of *Chlamydia* Dsb proteins, their substrates, and their interaction will help us to understand the critical yet incompletely characterized role of disulfide bonding in the *Chlamydia* infection lifecycle.

## Materials and methods

### Investigation of CtDsbA expression levels by Western blot

McCoy B (sourced from American Tissue Culture Collection (ATCC): CRL 1696, mouse fibroblast like immortalised cells) cells were cultured in 6-well plates at 300,000 cells per well in 3 mL of culture media (high glucose Dulbecco’s Modified Eagle Medium (DMEM; Sigma-Aldrich D6546) supplemented with heat-inactivated 10% foetal calf serum, 4 mM L-Glutamine,100 μg/mL streptomycin and 50 μg/mL gentamycin) and incubated at 37˚C with 5% CO2. 24 hours after seeding, cells were infected with *C*. *trachomatis* LGV-2 (strain 443/Bu, sourced from the ATCC: VR-902B) at a multiplicity of infection of 1. The infected cells were rocked for 30 mins to disperse infectious units, then centrifuged at 500 × g for 30 mins to synchronise the infection, prior to a media change at 4 h post infection (h PI) with the addition of 1 μg/mL cycloheximide. Uninfected controls were cultured in the same manner except non-infectious media was added at 24 h after seeding. Infected wells were harvested into SPG (10 mM sodium phosphate, 250 mM sucrose, 5 mM L-glutamate) via scraping at 20, 32 and 44 h PI, and uninfected wells were harvested at 44 h PI only. Two sets of triplicate wells were pooled for each sample and immediately added to 4x Bolt LDS Sample Buffer (Invitrogen; lithium dodecyl sulfate at pH 8.4) to give a final concentration of 1x sample buffer and 50 mM dithiothreitol (DTT), and boiled at 99°C for 20 mins to lyse. Independent experiments were repeated on three separate occasions. A representative example Western blot is shown in the results. For Western blot analysis samples were separated by SDS-PAGE using Bolt 4–12% Bis-Tris gels (Thermofisher) in MES SDS running buffer (50 mM MES, 50 mM Tris Base, 0.1% SDS, 1 mM EDTA, pH 7.3) at 200 V for 20 min. Following electrophoresis, proteins were transferred onto a nitrocellulose membrane using an iBlot 2 transfer device (Thermofisher). Proteins were probed with an anti-CtDsbA antibody from serum harvested from a rabbit immunised with purified recombinant CtDsbA protein (purchased from Osenses). The serum containing anti-CtDsbA antibody was prepared as a 1:400 dilution in 1% skim milk powder in TBS (50 mM Tris-Cl, pH 7.6, 150 mM NaCl). An anti-RpoB antibody (*Chlamydia* loading control, MyBioSource) was similarly prepared in a 1:20,000 dilution, and anti-MOMP (second *Chlamydia* loading control, Invitrogen) was prepared as a 1:5000 dilution. Anti-rabbit IgG, or anti-mouse IgG coupled with horse radish peroxidase were applied as a secondary antibody (1:10,000 dilution in 1% skim milk powder in TBS) and the final bound protein detected using enhanced chemiluminescence (Amersham ECL Prime Western Blotting Detection Reagent, GE Healthcare) and an Amersham AI600 imager (GE Healthcare).

### Investigation of CtDsbA protein by confocal microscopy

At 20, 32 and 44 h post infection, coverslip cultures of McCoy B cells infected with *C*. *trachomatis* LGV-2 were fixed with 4% PFA for 10 min. Cultures were stained with the anti-CtDsbA antibody used for the Western blot analysis, and a secondary goat anti-rabbit IgG antibody conjugated with Alexa Flour 488. Positive labelling controls were stained with anti-CtHtrA antibody raised in rabbit and the same secondary used against anti-CtDsbA. The host cell nucleus was stained with 4′,6-diamidino-2-phenylindole (DAPI), and the host cell cytoskeleton was stained with Alexa Fluor 594 Phalloidin. Coverslips were mounted on slides in Prolong Gold (Invitrogen) prior to immunofluorescence imaging on a Nikon A1 confocal LASER microscope.

### Expression and purification of CtDsbA and variants

The recombinant CtDsbA used in this study corresponds to residues 34–238 of *C*. *trachomatis* dsbA as reported previously [36]. CtDsbA-SSS is a variant in which each of three non-active cysteines are mutated to serine (C66S, C80S and C141S). CtDsbA and CtDsbA-SSS were expressed in *E*. *coli* BL21 (DE3) pLysS (Invitrogen) and purified as described in [36] with one modification; to improve stability of the protein the buffer was changed to 25 mM Tris pH 7.4, 150 mM NaCl. As required CtDsbA and CtDsbA-SSS were reduced and oxidised by incubation with 100-fold molar excess of dithiothreitol (DTT) or 100 fold molar excess of oxidised glutathione, respectively. The protein redox state was confirmed by Ellman’s reagent [56].

### Expression and preparation of membranes containing CtDsbB and variants

CtDsbB (Uniprot ID 084179) was identified by homology to EcDsbB (Fig 3). Additional CtDsbB variants in which pairs of cysteine residues in each of the periplasmic loops were mutated to serine were also designed: namely CtDsbB-SSCC (C36S and C39S in periplasmic loop 1) and CtDsbB-CCSS (C98S and C104S in periplasmic loop 2). The DNA for CtDsbB and mutants were purchased as gBlocks (Integrated DNA technologies) with a 5’ XhoI and a 3’ NdeI restriction site for insertion to a pET21a vector. This added a non-cleavable C-terminal His6 tag.

All constructs were expressed in *E*. *coli* C41(DE3) (kindly provided by Cy Jeffries, University of Sydney) cells using PASM 5052 autoinduction media [57] containing ampicillin and incubated at 30°C for 18–24 h with orbital shaking at 200 rpm. Cells were harvested by centrifugation at 6,000 rpm for 15 mins. Harvested cells were resuspended in 25 mM 2-(N-morpholino) ethanesulfonic acid (MES) buffer at pH 6, 150 mM NaCl and lysed using a cell disrupter (Constant Systems Ltd) (one passage at 28 KPsi followed by a second passage at 30 KPsi). Unbroken cells and debris were removed by centrifugation at 18,500 rpm for 30 mins. Membranes were harvested from the supernatant by ultracentrifugation (42,000 rpm for 1 hr) at 4°C. The pellet was resuspended in 25 mM MES pH 6, 150 mM NaCl using a glass dounce homogeniser.

For the model substrate folding assay, the amount of CtDsbB present in the crude membranes was quantified by SDS-PAGE analysis, in comparison to a series of known amounts of detergent solubilised purified EcDsbB (0, 0.5, 1, 2 and 5 μg). A series of dilutions of the membrane preparation were prepared in sample loading dye alongside EcDsbB standards, and densiometric analysis and comparison of the bands used to estimate the concentration of CtDsbB in the membrane preparations.

### Solubilisation and purification of CtDsbB

Crude membranes containing CtDsbB were solubilised in 25 mM MES pH 6, 150 mM NaCl with 0.5% DDM under vigorous stirring at 4°C for 1 h. Solubilised protein was isolated by ultracentrifugation for 1 h at 42,000 rpm at 4°C, and the supernatant loaded onto a 5 mL HisTrap^TM^ HP column (GE Healthcare) equilibrated in 25 mM MES pH 6, 150 mM NaCl with 0.015% DDM (Buffer 1). The column was washed with Buffer 1 plus 20 mM imidazole (10 x column volume (CV)), with sequential washes of 40 mM imidazole (10 x CV), 80 mM imidazole (10 x CV), 120 mM imidazole (5 x CV) and eluted with 500 mM imidazole (5 x CV). The eluted protein has a distinctive orange colour, suggesting a bound ubiquinone cofactor [48]. Protein was further purified by size exclusion chromatography using a Superdex^TM^ 200 16/60 column (GE Healthcare) in Buffer 1. Protein purity was evaluated by SDS-PAGE on a NuPAGE 12% Bis-Tris gel (Thermofisher) with MES running buffer (50 mM MES pH 7.3, 50 mM Tris, 0.1% sodium dodecyl sulfate (SDS) and 1 mM Ethylenediaminetetraacetic acid (EDTA)).

### Model substrate folding assay

The peptide oxidation assay was performed as reported in [36] except that crude membranes containing recombinant CtDsbB were used instead of oxidised glutathione to sustain CtDsbA activity. Briefly the assay was performed in a 384-well plate (Perkin Elmer, USA). A solution of 50 mM MES, 50 mM NaCl, 2 mM EDTA, pH 5.5, 8 μM of the DsbB and either 80 nM (EcDsbA) or 640 nM (CtDsbA or CtDsbA-SSS) were added to the wells in a total volume of 25 μL. Adding 25 μL peptide to a final concentration of 10 μM started the reaction. Change in fluorescence was monitored at excitation 340 nm and emission 615 nm, with a delay of 100 μs and read time of 100 μs, using a Synergy H1 Multimode plate reader (BioTek, USA). Plotted data shows mean and SD for two biological replicates.

### Redox state of analysis of reduced CtDsbA and CtDsbA-SSS in the presence of CtDsbB using gel-shift assay

Purified reduced CtDsbA or CtDsbA-SSS (15 μM) were mixed with equimolar amounts of detergent solubilised purified CtDsbB (15 μM) in the presence or absence of equimolar amounts of UQ1 (15 μM) in 25 mM MES pH 6, 150 mM NaCl, and 0.1% DDM in a total volume of 60 μL. 5 μL samples were taken immediately after adding CtDsbB (t = 0 mins), and then after 10, 20, 30, 60, 90 and 120 min incubation, before trichloroacetic acid (TCA) 10% w/v mediated precipitation, washing with ice-cold acetone, labelling with 4-acetamido-4’-maleimidylstilbene-2,2’-disulfonic acid (AMS) and SDS-PAGE analysis as described previously [36]. Control reactions included reduced CtDsbA/CtDsbA-SSS with UQ1 (15 μM), CtDsbA/CtDsbA-SSS with 5 mM oxidised glutathione (GSSG), and CtDsbA/CtDsbA-SSS with 5 mM DTT incubated for 2 hrs. Data presented is representative of three independent experiments from three different purifications of recombinant CtDsbA, CtDsbA-SSS and CtDsbB, expressed following independent transformations of *E*. *coli*.

### Relative stability of oxidised and reduced forms of CtDsbA-SSS

The thermal stability of reduced and oxidised CtDsbA-SSS was determined by circular dichroism using a Jasco J-810 circular dichroism (CD) spectropolarimeter. Measurements were carried out with 10 μM protein in 100 mM NaH_2_PO_4_/Na_2_HPO_4_, 0.1 mM EDTA, pH 7.0 in a 1 mm quartz cuvette. The unfolding was monitored as a change in molar ellipticity at 220 nm with a heat rate of 0.5 K/min from 298 K to 398 K. Plotted data shows mean and SD for two biological replicates.
